# Evaluating Care Interactions and Characteristics: The Modified Quality of Interaction Scale

**DOI:** 10.1093/geroni/igaf122.1338

**Published:** 2025-12-31

**Authors:** Rachel McPherson, Barbara Resnick, Sarah Holmes, Anju Paudel, Elizabeth Galik

**Affiliations:** University of Maryland School of Nursing, Baltimore, Maryland, United States; University of Maryland, Baltimore, Maryland, United States; University of Maryland School of Nursing, Baltimore, Maryland, United States; The Pennsylvania State University, University Park, Pennsylvania, United States; University of Maryland School of Nursing, Baltimore, Maryland, United States

## Abstract

While there is growing recognition of the importance of measuring care quality in long-term care settings, there has been limited focus on assessing care interactions. Existing measurement tools capture only a few aspects of these interactions. The Quality of Interactions Scale, an existing measure that examines characteristics (e.g., location and interpersonal distance during interactions) and quality (e.g., positive, negative, and neutral) of staff-resident interactions, was modified to assess a broader range of interaction characteristics, including staff and resident demographics, which may influence care quality. This study aimed to (1) test the reliability and validity of the Modified Quality of Interactions Scale (MQuIS), and (2) evaluate differences in the quality of care interactions (positive, neutral, or negative) based on staff-resident racial concordance (i.e., shared racial background) versus discordance (i.e., different racial background). Data was collected in four assisted living facilities, and the sample included 152 staff-resident interactions. Results showed strong reliability (item reliability = 0.98) and supported validity of MQuIS, with significant positive associations between active resident engagement and positive interactions. Care interactions were significantly more positive when staff and residents shared the same race (M = 5.0, SD = 2.0) compared to different racial backgrounds (M = 4.2, SD = 1.9), F(1,150) = 3.51, p = .032. These findings support the use of MQuIS to evaluate care interactions and highlight the importance of considering staff-resident demographics in assessments of dementia care and outcomes. Our findings also suggest that simple modifications to existing measures can help to capture and examine important aspects in dementia care and outcomes.

